# Bibliometric and visual analysis of coronary microvascular dysfunction

**DOI:** 10.3389/fcvm.2022.1021346

**Published:** 2022-11-15

**Authors:** Xiaoxiao Lin, Guomin Wu, Beibei Gao, Shuai Wang, Jinyu Huang

**Affiliations:** ^1^The Fourth School of Clinical Medicine, Zhejiang Chinese Medical University, Hangzhou, China; ^2^Department of Cardiology, Affiliated Hangzhou First People’s Hospital, Zhejiang University School of Medicine, Hangzhou, China; ^3^Department of Translation Medicine Center, Affiliated Hangzhou First People’s Hospital, Zhejiang University School of Medicine, Hangzhou, China

**Keywords:** coronary microvascular dysfunction (CMD), bibliometric analysis, visual maps, HFpEF, treatment

## Abstract

**Background:**

Coronary microvascular dysfunction (CMD) may play an important role in various cardiovascular diseases, including HFpEF and both obstructive and non-obstructive coronary artery disease (CAD). To date, there has been no bibliometric analysis to summarize this field. Here, we aim to conduct a bibliometric analysis of CMD to determine the current status and frontiers in this field.

**Materials and methods:**

Publications about CMD were taken from the Web of Science Core Collection database (WOSCC). WOSCC’s literature analysis wire, the VOSviewer 1.6.16, and CiteSpace 5.1.3 were used to conduct the analysis.

**Results:**

A total of 785 publications containing 206 reviews and 579 articles are included in the sample. The leading authors are Iacopo Olivotto, Paolo G. Camici, and Carl J. Pepine. The most productive institutions are the University of Florence, Cedars Sinai Medical Center, and Harvard University. The most productive countries are the USA, Italy, and England. There are a total of 237 journals that contribute to this field, and the leading journals in our study were the International Journal of Cardiology, the European Heart Journal and the JACC. From 2012 to 2021, the top three most-cited articles focused on the association between HFpEF and CMD. The important keywords are heart failure, hypertrophic cardiomyopathy, chest pain, women, coronary flow reserve (CFR), endothelial dysfunction and prognostic value. “Positron emission tomography” shows the strongest burst strength, followed by “blow flow” and “artery.” The keywords that started to burst from 2015 are particularly emphasized, including “heart failure,” “coronary flow reserve,” and “management.”

**Conclusion:**

Studies about CMD are relatively limited, and the largest contribution comes from the USA, Italy and England. More studies are needed, and publications from other countries should be enhanced. The main research hotspots in the CMD field include CMD in patients with HFpEF, sex differences, the new methods of diagnosis for CMD, and the effective treatment of CMD. Attention should be given to CMD in patients with HFpEF, and untangling the association between CMD and HFpEF could be helpful in the development of physiology-stratified treatment for patients with CMD and HFpEF.

## Introduction

Coronary microvascular dysfunction (CMD) is an underdiagnosed and underrecognized condition that is related to an increased incidence of adverse cardiac events (1–4). CMD could present as chest pain with imaging abnormalities and/or electrocardiographic changes evident in stress testing but without obstructive coronary artery disease on angiography. The risk factors for CMD are similar to those for coronary artery disease (CAD), including chronic inflammation, hyperlipidemia, hyperglycemia, hypertension, and aging (1, 5–7). CMD could be diagnosed by the use of non-invasive coronary assessment via PET, CMR, or echocardiography and invasive coronary assessment via doppler or thermodilution. The treatment of CMD encompasses antianginal medications including ranolazine, ivabradine, and nicorandil, anti-atherosclerotic drugs including statins, ACEIs, and calcium-channel blockers, and some non-pharmacological treatments including a well-balanced diet, lifestyle modifications, cognitive behavioral therapy (CBT) and enhanced external counterpulsation (5).

Coronary microvascular dysfunction can be divided into four types including dysfunction in the presence of obstructive CAD, dysfunction due to myocardial diseases, dysfunction in the absence of myocardial and CAD diseases, and iatrogenic dysfunction. CMD is prevalent across various cardiovascular diseases (CVDs) including non-ischemic cardiomyopathies, angina with and without obstructive CAD, takotsubo syndrome, myocardial infarction, and heart failure (HF). Among them, CMD in heart failure with HFpEF has drawn increasing attention. In Shah’s study, the high prevalence of CMD in HFpEF (75%) was demonstrated (8). Taqueti et al. found that impaired CFR in symptomatic patients without overt CAD was independently associated with adverse events such as HFpEF hospitalization (9). In specific populations, CMD may have a pathophysiological and prognostic role. Previous studies have shown that CMD may play an important role in different cardiovascular diseases including HFpEF, obstructive and non-obstructive CAD. To date, there has been no bibliometric analysis to summarize this field. We aim to conduct a bibliometric analysis of CMD to determine the current status and frontiers in this field.

## Materials and methods

### Search strategy

The WoSCC has been widely used for bibliometric analysis widely (10, 11). In our study, publications about CMD were taken from the WoSCC. The search term used was TS = “coronary microvascular dysfunction” OR “Coronary Microvascular Endothelia Inflammation” OR “Coronary Microvascular Rarefaction” OR “coronary microvascular endothelial dysfunction.” The search results were narrowed by publication data including a date range of: 1 January 2000 to 31 December 2021, a publication language of English and the article types of reviews and articles.

### Data collection and analysis

The VOSviewer 1.6.16 (Rotterdam, Netherlands), CiteSpace 5.1.3 (Philadelphia, PA, USA), and WoSCC literature analysis wire were used to conduct the our study’s analysis (12, 13). The *h*-index was used to evaluate the citations of the selected publications. In our study, VOSviewer1.6.16 software was used to determine co-authorship keywords, authors, countries/regions, and institutions. Publication year, document type, categories, the distribution of authors, country/region, institutions, and *h*-index were analyzed by the WoSCC literature analysis wire. CiteSpace 5.1.3 was used to the burst detection analysis of keywords. Total link strength (TLS) was used to evaluate the cooperation relationship.

## Results

### Publication output

**FIGURE 1:**
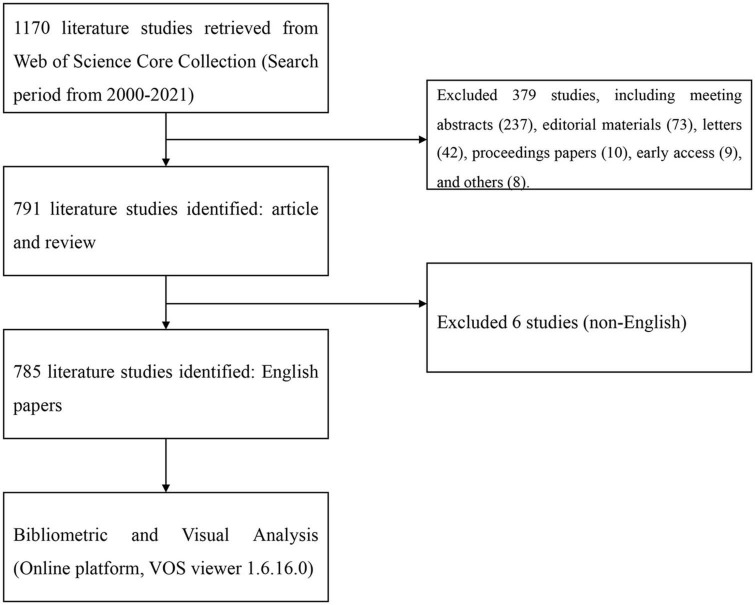
Flowchart of the inclusion and exclusion criteria.

**FIGURE 2:**
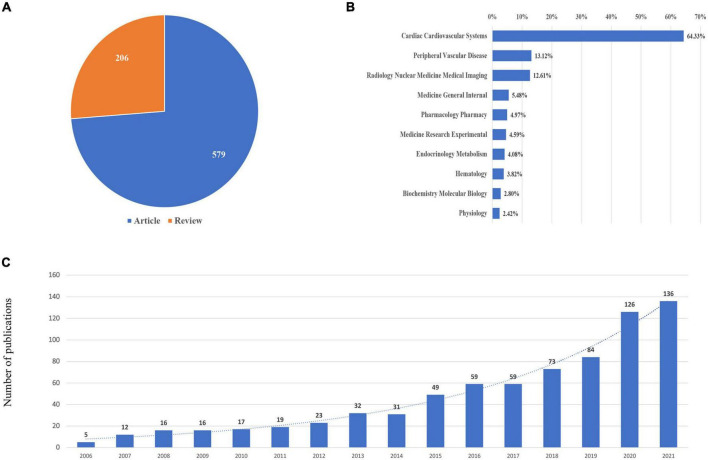
Yearly quantity and literature type of publications on CMD from 2000 to 2021. **(A)** Literature types distribution. **(B)** Subject categories distribution. **(C)** Annual publications quantitative distribution.

A total of 785 publications containing 206 reviews and 579 articles were included (Figure 1). The publications generally showed an upward trend from 2006. The major subject categories were cardiac cardiovascular systems (505 publications), peripheral vascular disease (103 publications), radiology nuclear medicine medical imaging (99 publications), medicine general internal (43 publications), and pharmacology pharmacy (38 publications), which is shown in Figure 2.

### Distribution of authors

**FIGURE 3:**
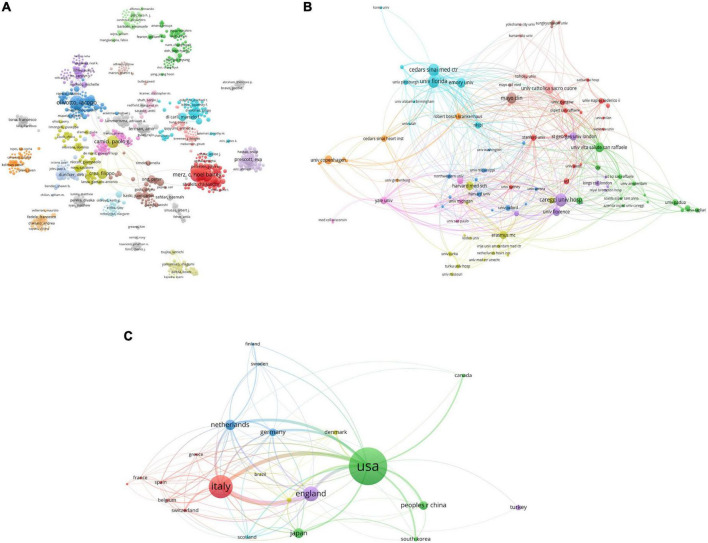
Visualization knowledge maps of the co-authorship. **(A)** The co-authorship map of authors which indicates the authors that cooperate in the field of CMD; **(B)** the co-authorship map of organizations. The institution with the strongest total link strength is the University of Florida. **(C)** The co-authorship map of countries. The number of collaborators with the USA is 300 and the total link strength is 230. Different colors indicate different clusters and the size of nodes indicates the number of publications. The thickness of the lines represents the link strength of the countries.

From this field, a total of 4,207 authors were included. Among them, Iacopo Olivotto was the leading author from Italy with 45 publications, followed by Paolo G Camici with 40 publications, Carl J Pepine with 32 publications, Janet Wei with 27 publications, and C Noel Bairey Merz with 26 publications. Paolo G Camici, Filippo Crea, and Iacopo Olivotto were the most highly cited authors. The uppermost h-index value was for Paolo G Camici (*h*-index: 29), followed by Iacopo Olivotto (*h*-index: 23), Filippo Crea (*h*-index: 15) and Amir Lerman (*h*-index: 15). The coauthorship map of authors is shown in Figure 3A. The top three coauthorship triads of authors were C Noel Bairey Merz (TLS = 238), Janet Wei (TLS = 196) and Carl J. Pepine (TLS = 175).

### Distribution by countries/regions and institutions

All publications were taken from 48 countries/regions and 1,064 organizations. The most productive institutions were the University of Florence (52 publications with 2,536 citations), the Cedars Sinai Medical Center (51 publications with 2,867 citations), Harvard University (50 publications with 2,654 citations), the Azienda Ospedaliero Universitaria Careggi (48 publications with 2,395 citations), and Imperial College London (42 publications with 4,510 citations). The coauthorship map of institutions is shown in Figure 3B. The top three institutions involved in coauthorship were the University of Florida, Careggi University Hospital and Cedars Sinai Medical Center. For countries/regions, the USA had the most publications (301 documents), followed by Italy (178 documents), England (111 documents), Netherlands (72 documents), and Japan (67 documents). The top three countries/regions involved in coauthorship were the USA, Italy, and England. The coauthorship map of countries/regions and the top 10 high-yield countries/regions, institutions and authors are summarized in Figure 3C and Table 1.

**TABLE 1:** Ranking of the top 10 authors, institutions, and countries based on publications.

Items	Publications					Co-authorship maps		
	Rank	Name	Number	Citations	H-index	Rank	Name	Total link strength
Country	1	USA	301	13,574	55	1	USA	230
	2	Italy	178	8,531	44	2	Italy	182
	3	England	111	6,985	38	3	England	149
	4	Netherlands	72	5,125	28	4	Netherlands	114
	5	Japan	67	1,343	19	5	Germany	77
	6	People’s Republic of China	58	477	12	6	Australia	61
	7	Germany	51	4,248	26	7	Japan	47
	8	Turkey	36	561	12	8	Scotland	39
	9	Denmark	31	565	12	9	Spain	34
	10	Australia	29	1,104	18	10	Belgium	32
Institution	1	University of Florence	52	2,536	26	1	University of Florida	105
	2	Cedars Sinai Medical Center	51	2,867	19	2	Careggi University Hospital	103
	3	Harvard University	50	2,654	24	3	Cedars Sinai Medical Center	92
	4	Azienda Ospedaliero Universitaria Careggi	48	2,395	24	4	Università Vita-Salute San Raffaele MILANO	84
	5	Imperial College London	42	4,510	26	5	Erasmus Medical Center	75
	6	Mayo Clinic	39	3,648	23	6	Yale University	67
	7	Brigham Women’s Hospital	38	2,461	23	7	St George’s University of London	66
	8	State University System of Florida	38	2,069	17	8	Imperial College London	60
	9	University of Florida	38	2,069	17	9	University of Michigan	59
	10	Catholic University of the Sacred Heart	32	3,391	18	10	Emory University	58
Author	1	Iacopo Olivotto	45	2,187	23	1	C. Noel Bairey Merz	238
	2	Paolo G. Camici	40	5,623	29	2	Janet Wei	196
	3	Carl J. Pepine	32	1,485	13	3	Carl J. Pepine	175
	4	Janet Wei	27	475	9	4	Puja K. Mehta	146
	5	C. Noel Bairey Merz	26	1,392	13	5	Chrisandra Shufelt	116
	6	Filippo Crea	24	3,177	15	6	Iacopo Olivotto	113
	7	Eileen Handberg	21	825	8	7	Galen Cook-Wiens	111
	8	Eva Prescott	21	408	9	8	Eileen Handberg	110
	9	Amir Lerman	21	1,027	15	9	Eva Prescott	110
	10	Puja K. Mehta	21	483	10	10	Daniel S. Berman	94

### Distribution by journal

**FIGURE 4:**
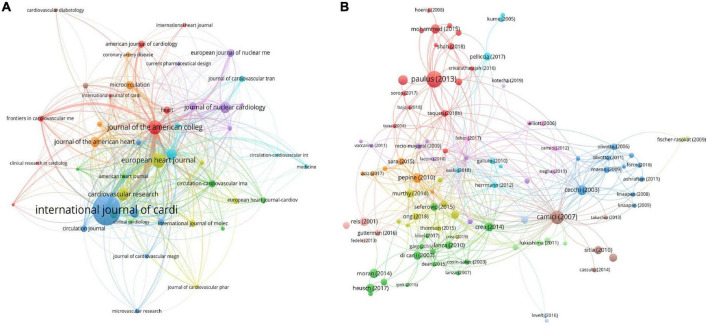
Visualization knowledge maps of citation. **(A)** Citation map of Journal; **(B)** citation map of documents. Different color indicates different clusters. The size of the nodes represents the counts of citations. The distance between the two nodes indicates their correlation.

A total of 237 journals contribute to this field. Among them, the International Journal of Cardiology was the leading journal with 55 documents, followed by EHJ with 25 documents and JACC with 23 documents. The coauthorship map of journals and the top 10 high-yield journals are summarized in Figure 4A and Table 2.

**TABLE 2:** Ranking of the top 10 journals based on publications.

Ranking	Journal name	Country	Counts	Citation	H-index
1	International Journal of Cardiology	Ireland	55	919	14
2	European Heart Journal	England	25	2,852	20
3	Journal of the American College of Cardiology	USA	23	4,934	20
4	Cardiovascular Research	England	20	885	16
5	Journal of Nuclear Cardiology	USA	19	192	8
6	Circulation	USA	18	3,390	18
7	Atherosclerosis	USA	15	339	10
8	Journal of The American Heart Association	USA	15	243	8
9	Microcirculation	England	13	174	6
10	American Journal of Physiology Heart and Circulatory Physiology	USA	12	373	9

### Analysis of high-cited references

The characteristics of publications were summarized in Table 3 (1, 14–32). The most-cited reference was published in the JACC and authored by Paulus W. J. and Tschope C. The second most-cited reference was published in NEJM and authored by Paolo G. Camici and Filippo Crea in 2007. The third most-cited reference was published in the JACC and authored by Piero O. Bonetti et al in 2004. To explore the hotspots in this field over recent years, we summarize the top three most highly cited articles in the last decade (2012–2021) The most highly cited article was published in the JACC by Paulus W. J. in 2013 (26). In this article, Paulus first proposed the association between CMD and HFpEF, which differs from that between HFpEF and HFrEF. Attention should be given to this new HFpEF paradigm of CMD with potential important diagnostic and therapeutic implications. The second most highly cited article was published by Shah S. J. in 2016 (31). Shah believed that CMD played an important role in HFPEF and proposed personalized therapeutic strategies. The third most cited article was published by Mohammed SF in 2015, who found that patients with HFpEF had coronary microvascular rarefaction compared with controls following autopsy. The citation map of documents is shown in Figure 4B.

**TABLE 3:** Ranking of the top 20 highest cited references.

Rank	Title	Journals	Total citations	Publication year	First author
1	A novel paradigm for heart failure with preserved ejection fraction comorbidities drive myocardial dysfunction and remodeling through coronary microvascular endothelial inflammation	JACC	1,739	2013	Paulus, WJ
2	Medical progress - Coronary microvascular dysfunction	NEJM	1,082	2007	Camici, PG
3	Non-invasive identification of patients with early coronary atherosclerosis by assessment of digital reactive hyperemia	JACC	713	2004	Bonetti, PO
4	Coronary microvascular dysfunction and prognosis in hypertrophic cardiomyopathy	NEJM	507	2003	Cecchi, F
5	Phenotype-specific treatment of heart failure with preserved ejection fraction a multiorgan roadmap	Circulation	488	2016	Shah, SJ
6	Coronary microvascular reactivity to adenosine predicts adverse outcome in women evaluated for suspected ischemia results from the national heart, lung and blood institute WISE (Women’s Ischemia Syndrome Evaluation) study	JACC	460	2010	Pepine, CJ
7	Coronary microvascular rarefaction and myocardial fibrosis in heart failure with preserved ejection fraction	Circulation	404	2015	Mohammed, SF
8	The global burden of ischemic heart disease in 1990 and 2010	Circulation	400	2014	Moran, AE
9	Coronary microvascular dysfunction: an update	European heart journal	383	2014	Crea, F
10	The pathophysiology of acute myocardial infarction and strategies of protection beyond reperfusion: a continual challenge	European heart journal	378	2017	Heusch, G
11	Coronary microvascular dysfunction is highly prevalent in women with chest pain in the absence of coronary artery disease: Results from the NHLBI WISE study	American heart journal	365	2001	Reis, SE
12	From endothelial dysfunction to atherosclerosis	Autoimmunity reviews	318	2010	Sitia, S
13	Role of chronic hyperglycemia in the pathogenesis of coronary microvascular dysfunction in diabetes	JACC	318	2003	Di Carli, MF
14	Effects of sex on coronary microvascular dysfunction and cardiac outcomes	Circulation	308	2014	Murthy, VL
15	Hemodialysis-induced cardiac dysfunction is associated with an acute reduction in global and segmental myocardial blood flow	Clinical journal of the American society of nephrology	305	2008	McIntyre, CW
16	Clinical diabetic cardiomyopathy: a two-faced disease with restrictive and dilated phenotypes	European heart journal	290	2015	Seferovic, PM
17	Ischemia and no obstructive coronary artery disease (INOCA) developing evidence-based therapies and research agenda for the next decade	Circulation	286	2017	Merz, CNB
18	Primary coronary microvascular dysfunction clinical presentation, pathophysiology, and management	Circulation	286	2010	Lanza, GA
19	Pathophysiology of takotsubo syndrome	Circulation	277	2017	Pelliccia, F
20	Coronary microvascular dysfunction: mechanisms and functional assessment	Nature reviews cardiology	239	2015	Camici, PG

### Analysis of keywords co-occurrence clusters

**FIGURE 5:**
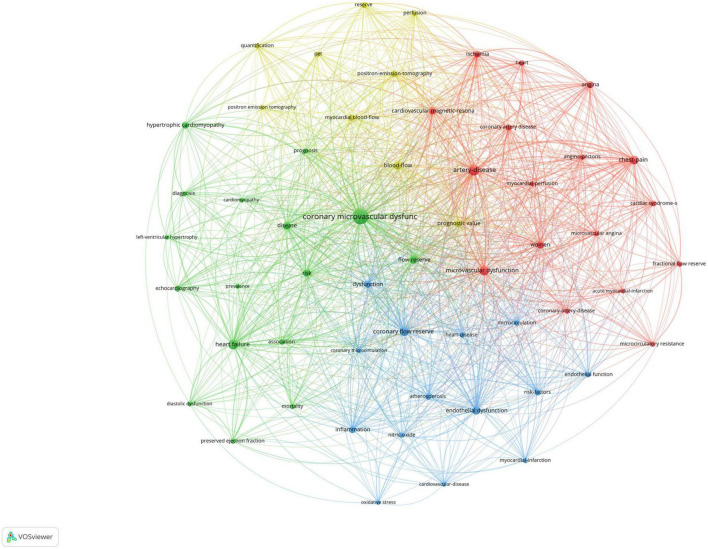
Visualization of keyword co-occurrence analysis. The size of nodes indicates the frequency of occurrences of the keywords. The lines between the nodes represent their co-occurrence in the same publication. The shorter the distance between two nodes, the larger the number of co-occurrence of the two keywords.

Figure 5 shows the co-occurrence map of keywords, and four research directions are also shown. The green cluster includes heart failure, hypertrophic cardiomyopathy, and disease. The red cluster includes artery disease, chest pain, women, and cardiac syndrome. The blue cluster includes coronary flow reserve (CFR), endothelial dysfunction, and inflammation. The yellow cluster includes blow flow, PET, and prognostic value.

### Burst detection of keywords

**FIGURE 6:**
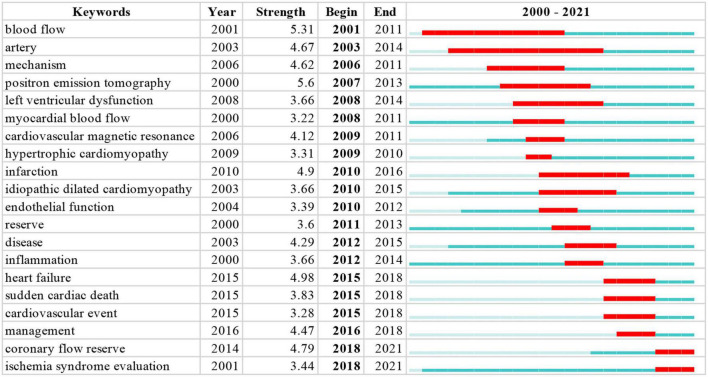
Top 20 Keywords with the strongest citation bursts. The blue indicates the timeline. The red segment indicates the start year, end year, and duration of the burst.

CiteSpace 5.1.3 is used to perform burst detection of keywords. The burst detection analysis of keywords can predict frontier research directions, reflect emerging academic trends, and display the hotspots in a field. Twenty prominent words were obtained by the burst detection analysis. “Positron emission tomography” shows the strongest burst strength, followed by “blow flow” and “artery.” The keywords that started to burst from 2015 are particularly emphasized, including “heart failure” (burst strength 4.98), “coronary flow reserve” (burst strength 4.79), and “management” (burst strength 4.47), which is shown in Figure 6.

## Discussion

### General information

To the best of our knowledge, this is the first bibliometric study in the field of CMD. A total of 785 publications are included. The leading authors are Iacopo Olivotto, Paolo G Camici and Carl J Pepine. The uppermost h-index value is for Paolo G. Camici, followed by Iacopo Olivotto, Filippo Crea, and Amir Lerman. The top three authors involved in coauthorship are C. Noel Bairey Merz, Janet Wei and Carl J. Pepine. All publications are taken from 48 countries/regions and 1,064 organizations. The most productive institutions are the University of Florence, the Cedars Sinai Medical Center, and Harvard University. The most productive countries are the USA, Italy, and England. The top three institutions involved in coauthorship are the University of Florida, Careggi University Hospital and Cedars Sinai Medical Center, and the top three countries/regions involved in coauthorship are the USA, Italy, and England. A total of 237 journals contribute to this field, and the leading journals are the International Journal of Cardiology, EHJ and JACC. From 2012 to 2021, the top three most-cited articles focused on the association between HFpEF and CMD. The important keywords used in this study are heart failure, hypertrophic cardiomyopathy, chest pain, women, CFR, endothelial dysfunction and prognostic value. “Positron emission tomography” shows the strongest burst strength, followed by “blow flow” and “artery.” The keywords that started to burst from 2015 are particularly emphasized, including “heart failure,” “coronary flow reserve,” and “management.”

Most of the included publications come from the USA, Italy, and England (590/785, 75.2%). Of the top 10 productive authors, six were from the USA, and the remaining four were from Italy. Of the top 10 institutions, 6 were from the USA, 3 were from Italy, and 1 was from England. Among cooperative relationships of authors, institutions and countries/regions, the USA, Italy and England were also prominent. The prominent countries in this field were again the USA, Italy, and England. Studies from and cooperation among other countries should be enhanced.

### Hotspots and frontiers

In accordance with the most cited references and the important keywords, the research frontiers and hotspots were found to be as follows: (1) The first relates to sex difference among CMD patients. Among the most-cited references, three explored sex differences in CMD patients (25, 28, 29). The keyword of women was in the red cluster. In the Women’s Ischemia Syndrome Evaluation (WISE) study (29, 33, 34). The keyword “women” occurred in the red cluster. The results of the Women’s Ischemia Syndrome Evaluation (WISE) study demonstrated that CMD was present in approximately 50% of women with NOCAD and chest pain. Murthy et al showed that CMD was highly prevalent in both men and women who were at-risk, and was associated with adverse outcomes irrespective of sex (25). Kobayashi et al. found that the microvascular function was similar in men and women (35). Recently, Chandramouli et al found that the drivers of CMD may differ by sex, but the prevalence of CMD in HFpEF was similar in women and men. They demonstrated that fibrosis and derangement in ventricular remodeling potentially predominate in women while the inflammatory paradigm of CMD seems to play a more important role in men (36). Based on recent evidence, the prevalence of CMD were similar in both women and men, but the mechanisms might be different, which should be considered in future therapeutic interventions. (2) The next hotspot involves the new methods of diagnosis for CMD. Among the most-cited references, four explored the diagnosis of CMD (1, 14, 15, 17). The keywords of coronary flow reserve, blow flow, PET, and prognostic value occur in the blue and yellow clusters. The diagnosis of CMD is usually made by the measurement of CFR or IMR with invasive coronary assessment via Doppler or thermodilution or CFR with non-invasive coronary assessment via PET, CMR, or echocardiography (37). the approaches of diagnosis for CMD are limited, and new methods are needed. A recent systematic review and meta-analysis showed that CFR was related to the rate of major adverse cardiovascular events (MACE) and all-cause mortality, and suggested that, CFR should be measured more routinely in clinical practice (38). In addition, coronary flow velocity reserve (CFVR) as assessed by echocardiography to evaluate the CMD is feasible and could predict the adverse outcomes in women with no obstructive CAD and angina (39). More diagnostic approaches are being developed. (3) The next hotspot involves the effective treatment of CMD, which was explored by five of the most cited references (15, 17, 20, 22, 31). The treatment of CMD includes the use of antianginal drugs, anti-atherosclerotic medication, and new therapeutic strategies such as phosphodiesterase-5 inhibitors and ivabradine (40–43). It is necessary to explore more effective treatments for CMD in further studies. In this regard, it is essential to distinguish between endothelium-independent and endothelium-dependent CMD since they may require different treatments. (4) The final hotspot involves CMD in HFpEF. Among the most-cited references, three explored the association between CMD and HFpEF (23, 26, 31), and all top three most-cited articles over recent 10 years were about it (23, 26, 31). The keyword of heart failure occurs in the green cluster. In 2013, the association between CMD and HFpEF was first proposed. In 2016, Kato et al conducted the first study and showed that the prevalence of CMD diagnosed with non-invasive coronary assessment was 76%, while Dryer et al demonstrated that the prevalence CMD diagnosed with invasive measurement in patients with HFpEF was 73.4% (44, 45). The largest multi-national study (PROMIS-HFpEF) showed that the prevalence of CMD was 75% (8). In 2022, a study conducted by Arnold et al demonstrated that the prevalence of CMD was 70% in patients with HFpEF, and it was the first study to explore the association between the CMR-measured indexes of microvascular function and clinical outcomes (46). several studies have explored the prevalence and correlation of CMD in patients with HFpEF. The prevalence of CMD was high in patients with HFpEF (over 70%) (8, 46–49). At present, all large-scale clinical trials had neutral results regarding the efficacy of pharmaceutical treatments for HFpEF with the exception of SGLT2 inhibitors (50–75), and even the effect of empagliflozin was attenuated in patients with an ejection fraction >65% (76). The heterogeneity of HFpEF may be the reason behind the failure of previous clinical trials to determine effective treatment for patients with HFpEF. Some patients could benefit from certain interventions, while others might not benefit or even be harmed by the same treatment. When these patients are mixed and treated together, it is difficult to identify an effective treatment. Untangling the association between CMD and HFpEF may be helpful to the development of physiology-stratified treatment for patients with CMD and HFpEF (CMD–HFpEF), to improving their life quality and to their prognosis. At present, none of the RCTs on HFpEF have been conducted in consideration of the presence of CMD. Interventions for HFpEF based on CMD may be effective. Targeted interventions may be effective for different types of patients with HFpEF according to microvascular function. For example, patients with impaired endothelium independent CMD may benefit more from soluble guanylate cyclase stimulators and phosphodiesterase inhibitors (PDEs). Patients with impaired endothelium-dependent CMD, on the other hand, may benefit more from inorganic nitrates that target the NO-PKG pathway of endothelial cells (77–79).

There are some limitations in our study. First, all publications were taken from the WoSCC as it is the best citation-based database, potentially resulting in bias and incompleteness. Second, only publications in English were included. Additionally, the literature information in 2022 was not counted due to the time of extraction.

## Conclusion

The studies about CMD are relatively limited, and the most prominent contribution is from the USA, Italy, and England. More studies are needed, and publications of other countries should be enhanced. The main research hotspots in the CMD field are CMD in patients with HFpEF, sex differences in CMD patients, the new methods of diagnosis for CMD, and the effective treatment of CMD. Further attention should be given to CMD in patients with HFpEF, and untangling the association between CMD and HFpEF may be helpful to the development of physiology-stratified treatment for patients with CMD and HFpEF.

## Data availability statement

The raw data supporting the conclusions of this article will be made available by the authors, without undue reservation.

## Author contributions

XL, GW, and BG were responsible for data collection, investigation, figures and tables construction, and writing the original draft. JH and SW contributed to the discussion and final review and editing. All authors reviewed and edited the final manuscript.
